# Migrant unaccompanied minors

**DOI:** 10.1016/S2352-4642(21)00194-2

**Published:** 2021-08-18

**Authors:** Susanna Corona Maioli, Jacqueline Bhabha, Kolitha Wickramage, Laura C N Wood, Rochelle Burgess, Vasileia Digidiki, Ludivine Erragne, Omar Ortega García, Robert Aldridge, Delan Devakumar

**Affiliations:** Institute for Global Health, UCL, 30 Guilford St, London, UK; Harvard FXB Center for Health and Human Rights, Harvard University, Boston, USA; UN Migration Agency, Migration Health Division, Global Migration Health Research and Epidemiology Unit, Manila, Philippines; Centre for Child & Family Justice Research, Lancaster University; Institute for Global Health, UCL, 30 Guilford St, London, UK; Harvard FXB Center for Health and Human Rights, Harvard University, Boston, USA; Médecins Sans Frontières, Paris, France; Local Integration Coordinator, CAFEMIN, Mexico City, Mexico; Institute of Health Informatics, UCL, London, UK; Institute for Global Health, UCL, 30 Guilford St, London, UK

## Abstract

‘Unaccompanied minors’ (UAMs) are a group of migrants whose global numbers are increasing. However, little is known about UAMs as data is not systematically collected in any region, if collected at all. UAMs are a diverse group, potentially at additional risk of harm to their health and integrity because they lack protection usually conferred by a family, which can lead to both short- and longer-term health impacts. This review summarises the most recent evidence regarding all the aspects relevant to UAM international migration and health. UAMs are entitled to protection that should follow their ‘best interests’ as a primary consideration but instead detention, sometimes under the guise of protection, remains a widespread practice. There is evidence of good long-term outcomes if these minors are provided with appropriate forms of protection, including health and psychosocial care. Instead, hostile immigration practices persist, which are clearly not in their best interests.

## Introduction

The Convention on the Rights of the Child (CRC) defines an unaccompanied minor (UAM) as a child or adolescent under the age of 18, who is “separated from both parents and other relatives and is not being cared for by any other adult who, by law or custom, is responsible for doing so”(1). Children who are accompanied by other adult caregivers, but not by parents, are defined as ‘separated children’ and form a distinct legal category. As many of these children and adolescents escape from deprivation, isolation and neglect, independent child migration *per se* can be a viable, life-saving strategy if it leads to opportunities that allow them to thrive(2). However, as travel routes become restrictive, migration can threaten the physical and emotional integrity of UAMs, especially if female, disabled or identified as LGBTQ+. Border enforcement with few and lengthy legal pathways to migrate, lead many UAMs into the hands of smugglers, risking highly unsafe travel and increasing their vulnerability to human trafficking(3–6). The credibility of many minors’ asylum claims is questioned without adequate legal assistance, coupled with deportation threat on becoming adult(7–9). This leads to disappearances from overburdened and unsuitable child protection systems(10) (see panel 1). The COVID-19 pandemic has made these pushbacks worse(11,12). For example, the public health emergency has been used by Hungary to justify closure of asylum procedures(13) and the US Customs and Border Patrol has reportedly deported UAMs despite credible asylum claims(11). Additionally, services to support UAMs are inadequate despite advocacy of existent recommendations, due to lack of political will(14). This leads to increasingly complex and challenging risks to child health, as exemplified by situations of indefinite containment such as the Greek islands of the Aegean Sea(15). This review summarises the health needs of UAMs. Demographic trends in UAM international migration and the risk factors they face are presented first, to better contextualise the evidence-based summary of physical and mental health outcomes that can aid professionals in providing the best care. We then address the laws and policies that can be implemented to reduce harm to UAMs. We discuss the implications of these findings in the conclusions and, finally, ways to address these implications in the recommendations section.

## Demographic trends

To acknowledge a problem, it is first important to document it and have adequate data to inform policy or advocacy. This is the first challenge regarding UAMs and their health. Trends in global UAM migration are mostly based on asylum applications filed by these minors. This not only means that numbers are likely to be large underestimates, but also that the more hidden populations -those undocumented or in transit– and subpopulations -females, LGBTQ+, disabled people- are mostly invisible. Collecting data on UAMs is complicated by the avoidance of detection that characterises irregular migration and the difficulty in identifying under-age migrants, given the common lack of identity documents and the inaccuracy of age assessment procedures(16). However, even when identified, varying definitions hinder comparability between regions(17) and estimates are often based on stock – the number at one point - rather than flow – the changes over time - figures; the latter being more useful for policy making and monitoring(18). Lack of adequate data is not only due to practical difficulties. There is a clear correlation between accurate data collection, State accountability and public perception of migration, especially regarding data on migrant children and migrant deaths(17,19).

While we do not have good data on UAMs, it is still important to provide a quantitative and geographical frame to UAM migration and its implications, for example child labour, trafficking and asylum claims. We will first describe available data on general child migration. The global number of migrant children was 33 million in 2019, an increase in absolute numbers from 24 million in 2000(20). As the world population has increased from 6 billion in 2000 to 7·7 billion in 2019(21), the proportion of migrant children to world population has remained constant at 0·4%. In 2019, children internally displaced -mainly in developing countries such as Brazil, China and Indonesia(22,23)- stood at 19 million, while 13 million were refugees(24,25), for whom age-disaggregated data is available. Regarding UAMs, according to the latest data available from UNICEF and UNHCR, 300,000 migrant children were reported to be unaccompanied or separated in 2015-2016(3,26), and 153,300 UAMs were reported among the refugee population in 2019(25). Due to border closures as a result of the COVID-19 crisis, the number of UAMs is likely to increase(27–29).

There is need of global estimates of child labour among migrant children and/or UAMs(30,31). However, there are documented links between migration, child labour and trafficking(22,32,33). In 2016, 152 million children between 5 and 17 years were working, of whom 48% (73 million) in hazardous labour(34). Children in need are at greater risk of being trafficked. Globally, 30% of all human trafficking victims are minors, according to the 2018 UN Office on Drugs and Crime report on trafficking in persons. Of these, 76·6% are girls(35). Although estimates of specific forms of trafficking to which migrant children and/or UAMs are most exposed are lacking(30,31), girls are likely to be at much higher risk.

Six main migration corridors situate UAM migration geographically: the Central Mediterranean route from North Africa to Italy through Libya(3); the Balkan route from Afghanistan, Syria and Iran to Serbia and Greece through Turkey(6); from Central America to the United States through Mexico(3); internal and across border displacement in the Horn of Africa(3,36); internal and across border labour migration in Southeast Asia(37,38); and from Afghanistan, Bangladesh and Myanmar to Australia through Malaysia, Thailand and Indonesia(39,40). The routes to Europe and North America are the ones with greater data availability, specifically data on asylum claims or apprehensions(3). For example, in the 27 EU countries and the UK, UAMs filed 17,110 asylum applications in 2019(41), mostly in the UK (3,651 applications), Greece (3,300), Germany (2,700) and Belgium (1,200)(42,43). This follows a decreasing trend from a peak of 95,205 applications filed in 2015, partly due to the EU-Turkey agreement on closure of the Balkan route in March 2016(44) and the Italy-Libya Memorandum of Understanding in February 2017(45). However, this number reflects asylum claims, rather than border crossings or detention numbers. UAMs continue to come smuggled and/or trafficked undetected, or else are pushed back, sometimes violently, even if they express the desire to claim asylum(6). Border closure is a sadly repeated policy in the Central America-Mexico-United States route, with the negotiated militarisation of Mexican and Guatemalan borders by both the Trump and Biden administrations(46,47). The recent increase in UAMs in this route parallels the Southwest border peak of 76,020 UAMs apprehended in fiscal year (FY) 2019(48,49).

Gender-based differences in child migration are important, although little is known. Studies from African countries report that girls are more likely to migrate internally in that region(50,51), while UAMs in international migration are mostly males, 15-17 years old(16,52). Data on LGBTQ+ child migration patterns is worryingly lacking.

Finally, the most common nationalities of UAMs are Afghanistan and Eritrea, based on global asylum applications(25); and Honduras, Guatemala and El Salvador for the Mexico-US route, for which only Border Patrol apprehension data is available(48). As of 2019, most unaccompanied child refugees were in Ethiopia (41,500), Uganda (40,000), Kenya (10,700) and Cameroon (9,000)(25).

In summary, we can see – despite lack of comprehensive data – how UAM migration is a global phenomenon and how UAMs are not a homogeneous population.

## Risk factors

This section will describe an overview of the health risks UAMs face by being underage unaccompanied migrants, keeping in mind the risk will impact differently on different ages, genders, abilities and geographical contexts.

UAMs are more vulnerable to migration risks and suffer different consequences compared to adults. The sensitive physical, mental and psychological developmental stages of childhood and adolescence enhance their risk of disease-and trauma, heightened by isolation from a protective family unit(53). While UAMs engage in survival, they are also being robbed of the time for developing their full potential through educational or vocational opportunities. The pre-existing life experiences and health status add to the diversity of UAM health impacts.

### Home country

Risks minors encounter in their home countries include poverty, extreme weather events, hunger, unhealthy living conditions, lack of health and education services, military or gang recruitment and direct or witnessed conflict, violence and abuse(53–56). Often, these risks come together and they are exacerbated for children who become separated from their family in the home country or who have family contexts of neglect and deprivation. Situations of poverty and hunger can lead to conflict and lack of services, or vice versa. The number of countries experiencing conflict is at its highest point since 1990, with protracted conflicts in Syria, Yemen and the Central African Republic(55). Other situations of violence in areas not described as conflict areas may cause the same devastating effects: gang violence in Central America and terrorist incursions in West and Central African countries cause disruption of education, abuse and death at unsustainable levels(57). All these risks are exacerbated by natural disasters(55). For example, the COVID-19 pandemic significantly exacerbated cases of intimate partner violence (IPV), gender based violence (GBV) and domestic abuse(58). Although not all UAMs have disrupted family structures, many experience or witness violence in their families(59–61), which may lead to the neglect or abuse that ultimately leave migration as means of escape. Despite the important risks of migration, it is sometimes riskier to stay; impacting those less able to migrate due to disability, illness or lack of resources(62).

### Transit

Migration transit has become increasingly risky as UAMs are faced with border closures such as described above(3,57). These ‘closures’ lead to increased smuggling and risk along the same route, or to prolonged confinement of UAMs in dangerous transit countries or conflict-laden areas(44,63). Complicated and long legal routes to rejoin family or receive asylum leave the option of being smuggled as a paradoxically easier option(3,56). Despite migration journeys being longer – sometimes months or years(56) – and riskier, profit-driven smuggling services to reach or move within the EU alone generated 4·5-5·7 billion Euro in 2015(3). Children may be exposed to considerable deprivation, exploitation, extortion, abuse and death during illegal journeys(64–66). The Missing Migrants Project (MMP) of the International Organization for Migration keeps a record of deaths of migrants in transit, from either remains or survivor reports. While not disaggregated by accompaniment status, from 2014 to 2018, the MMP recorded 678 child deaths by drowning in the Mediterranean, 40 while travelling by foot or land transport to Europe, 337 while migrating in Africa, 363 in Southeast Asia and 84 in the Mexico-United States route(67). Due to detection avoidance, fear of reporting and environmental degradation of remains, many fatalities remain unidentified(17). Many minors strive to remain under the radar: being exposed means losing independence, thus failing to meet responsibilities and expectations(54).

UAMs are also at heightened risk of being trafficked, often through deception, coercion and/or force. Whilst smuggling is a crime against the state, trafficking is a crime of severe human rights violation, with children relocated or harboured for the purposes of sexual, labour or criminal exploitation(6). The risk of sexual abuse in transit is particularly heightened for girls(56,66).

### Destination

In destination countries, there is often tension between border enforcement, which criminalises irregular migration, and a child rights-based assessment of health needs(56). Reports of physical, verbal and sexual abuse from border enforcement officials(3,66,68) are coupled with detention conditions in explicit violation of human rights(56,68). Prolonged detention is justified on the grounds of unavailable child welfare spaces, ‘flight risk’ or age determination dispute, with conditions such as sleep deprivation, inadequate food or water and denied medical care(69). Interviews with asylum officers can be scary and confusing, with recognition as a minor depending on age confirmation(70,71). Minors may hide their age because they want to continue the journey unhindered, as an agreed or threatened component of the smuggling arrangement or trafficking situation, or to hide their vulnerability(6). Further, due to long waiting times to obtain an immigration status and the cessation of their rights as children when they turn 18(4), many UAMs are left undocumented or in a state of legal limbo and disempowerment(71). It is difficult if not impossible to build a future under constant deportation threat: deportation carries the risk of returning to dangerous situations, with feelings of failure and peer discrimination(72). This rights chasm leads to a high number of disappearances(3), often into traffickers’ hands. For example, a European Migration Network 2020 report stated that 8,229 UAMs went missing in 2018 and 2019, in Italy and Greece alone(73).

UAMs are also exposed to discrimination, particularly if they are identified as Indigenous, Black, gender nonconforming or other minoritised groups(74–76). Media coverage which over-simplifies and dehumanises migration contexts promotes public views of migrants as a national threat, garnering support for border enforcement measures(69). Finally, while the risk of substance abuse among UAMs has not been vastly studied, it has been acknowledged as a pattern especially among refugee and asylum seeking UAMs(77–79). Many situations these minors find themselves in on arrival predispose to further community, gender based or sexual violence, including in reception facilities(80–85). Without access to a safe environment and with chronic uncertainty about the future, substance abuse becomes a coping mechanism(77,79,86) and it may also be used to control children in a trafficking context(87).

These significant risks in all phases of migration have an impact on children’s health and wellbeing, which makes the case for the urgency to increase awareness among child health practitioners. The following section describes health conditions specific to UAMs, which inform an evidence-based approach to their care.

## Health

UAMs are entitled to the “highest attainable standard of health” and to “non-discrimination” under the UNCRC(1). As such, the approach to UAM health should be understood within the wider frame of child and adolescent health and its impact on long-term and intergenerational health(88). An umbrella review of the health literature on UAMs can be found in Table 1, consisting of 8 reviews(89–96). This was updated to identify more recent literature: a summary of recent original articles not included in the reviews can be found in the Appendix. Based on this review, needs specific to this population include a mental health, developmental and nutritional assessment; infectious disease screening; examination of eyes, ear, nose, throat and teeth; and evaluation of serum lead levels(97). Special consideration must be given to the higher risk among this population of being victims of violence, sexual abuse and/or trafficking. This implies knowledge about child welfare procedures and careful evaluation of history and background of these minors to assess not just their health, but also their safety. A culturally sensitive and trauma-informed approach is mandatory when examining for signs of child abuse or violence(98). Thus, building trust is essential, especially among children who have received orders to stay away from, or have had negative experiences with, authorities. If possible, assessments should be made on multiple visits(97).

### Physical health and development

The most prevalent physical conditions reported in UAMs are: nutritional deficiencies; intestinal, respiratory and skin infections; low vaccination coverage and physical trauma – due to violence or harsh migration transit. Due to an unstable lifestyle and/or unhygienic living circumstances, dental caries has been described among UAMs with prevalence as high as 65%(91), which makes it important to include a dental examination during a medical review of UAMs(99–101). In a meta-analysis by Baauw et al(90), vitamin D deficiency was reported at 45% prevalence among refugee children from Africa, Asia and the Middle East, while iron-deficiency anaemia prevalence ranged from 4% to 18% in a review of undocumented children in Europe(91). While situations of conflict and displacement expose UAMs to undernutrition risk, more stable but uncertain living conditions predispose to becoming malnourished due to an unhealthy lifestyle and diet(89): risk factors for non-communicable diseases (NCDs)(102). Communicable disease prevalence is related to conditions during and after migration(91). Baauw et al(90) reported prevalence of 31% for intestinal infections and 11% for latent tuberculosis (TB) cases. This is in line with other studies reporting 44% prevalence of parasitosis among 226 UAMs in Geneva(103); 23% positive TB cases among 238 UAMs and 16% positive schistosomiasis cases among 163 UAMs from two UK clinics(104). Skin infections are also common: scabies prevalence was reported at 14% among 890 UAMs(105) and 30% among 154 UAMs(100) in two German studies. Constant movement, disruption in their countries of origin and emergencies are plausible reasons for the low vaccination coverage or knowledge of coverage described among UAMs(100,106). Importantly, among vaccine preventable diseases, only Hepatitis B virus has been frequently reported in UAMs – for example, 7·7% of 776 UAMs from high-prevalence countries tested positive in a German cross-sectional study(105). Screening for immunisation to measles, mumps, rubella, tetanus and diphtheria is recommended when vaccination history is uncertain(101,106). Physical trauma is common during illegal crossings; resulting in cuts, tendon lacerations, fractures and muscle contusions, which can leave long-term physical impairment(84,99).

Finally, although prevalence of sexually transmitted infections (STI) and unwanted pregnancies is unreported in UAMs, they are likely at increased risk due to both sexual exploitation vulnerability and higher risk-taking behaviour of adolescence. This means routine pregnancy testing and STI screening are relevant. Asymptomatic HIV and chlamydia are the most likely to occur in adolescents(107), while long-term consequences of sexual abuse include incontinence, infertility and sexual dysfunction(85,107).

### Mental health and development

Mental health conditions, such as post-traumatic stress disorder (PTSD), depression, anxiety, substance use, internalising and externalising behaviour, social withdrawal and stress are described extensively in UAM literature(89,92,95,96,108–111). Higher prevalence of PTSD is reported among unaccompanied compared to accompanied minors(92), with a German study describing a 28% prevalence difference(112). Some studies report higher prevalence among females and older ages. For example, female UAMs were reported to have an OR of 1.64 (p<0·1) of developing PTSD compared to their male counterparts(94). High prevalence of mental health conditions among UAMs is in line with evidence linking social adversity to poor mental health, making screening important to identify need of treatment and follow-up. Traumas that occur at younger ages may have lasting consequences, due to neurobiological effects in a developmentally immature brain(53,86) and to the impact of poor mental health on educational and personal development. Adolescence -the age period of most UAMs-is critical in terms of mental development, as brain maturation is shaped by interaction with the environment(113–116). UAMs may also develop considerable positive coping skills along migration that can contribute to promoting their wellbeing once in the context of safety. As such, priorities for UAM mental health and development support should emphasise safe recovery, assessment of complex social needs and prompt psychosocial care. Such efforts acknowledge vulnerabilities and resilience along a process of ensuring young people’s enjoyment of-their full rights.

### The intersection between adverse childhood experiences, protective childhood experiences and social determinants of health

Forced migration and violence can impact children’s mental and physical health through response to toxic stress, among other mechanisms(62). Toxic stress consists of both individual, stressful experiences a child goes through – described as Adverse Childhood Experiences (ACEs)(117)– and the child’s broader socio-economic context – described as Social Determinants of Health (SDH)(118). As described in the risk factors section, each phase of migration implies risk of ACEs such as emotional, physical and sexual abuse and neglect(117). ACEs specific to migration include loss, chronic uncertainty, discrimination, acculturative stress and lack of basic social, material and legal security(89,114). UAMs are highly likely to have experienced more than one ACE, possibly enhancing the relative risk of complex effects(62,108,114). This is coupled with the frequent context of structural racism and adverse SDH UAMs are in: they will often miss educational or vocational opportunities, without economic stability or protective social networks. Broader structural determinants of health, which impact children and adolescents more than other populations, are climate change and pervasive inequalities(118).

Mental and physical health consequences associated with experiencing toxic stress include alcoholism, drug abuse, depression, suicide attempt and higher risk of NCDs during adulthood, including ischemic heart disease and premature mortality(62,117,119). Mechanisms of these health outcomes include prioritising income generation over health-seeking behaviour(24), substandard accommodation, problematic access to healthcare and enduring nervous, endocrine and immune system changes; termed allostatic load(120).

While these circumstances increase UAMs’ vulnerability, particularly during crises such as COVID-19(24,95), it is important to emphasise the resilience of UAMs. Studies have identified important protective experiences, which we define as Protective Childhood Experiences (PCEs): community feeling, belonging, appropriate accommodation, psychosocial care, health and educational schemes(109). Individual traits are important coping mechanisms: faith, spirituality, sense of purpose and responsibility towards left behind family(53). Experiences that enhance these traits include having frequent contact with family and space to express religious beliefs. PCEs have an important role as they can determine a ‘physiological’ response to adversity, resulting in resilience(121). Thus, the social context and support an UAM finds is far from secondary and can have long lasting benefits (figure 1).

To support UAMs and advocate for their better health, it is important to understand the international legal framework that protects them, described in the next section.

## Legal implications

UAMs are entitled to legal protections set out in international treaties. How these treaties are applied in domestic provisions varies, even for countries that have ratified international legal obligations. Panel 2 gives examples of two countries – Uganda and Turkey – which have and have not, respectively, integrated international treaties in their national policies.

These international legal instruments establish a wide spectrum of responsibilities owed by states towards UAMs, and all children, on their territory. The central instrument governing children’s rights is the 1989 UNCRC, which consolidates into one treaty a wide range of human rights as they apply to children. It has been ratified by every member state of the United Nations except the United States and, as a result, has extensive applicability. While we appreciate the risks faced by young adults and the debates around the period of adolescence, for the purposes of this review we will use the CRC definition of ‘child’. This definition as ‘every human being below the age of eighteen years unless under the law applicable to the child, majority is attained earlier’(122) provides a unifying principle for approaching UAMs’ needs, irrespective of nationality. However, where legal identification is non-existent or questionable, adolescent UAMs are subjected to unreliable age determination tests(62) (see panel 1). It is important to acknowledge that a child is primarily a child, before any definition, and that transitioning to adulthood may imply protection needs that extend beyond age 18.

Other CRC provisions include non-discrimination against a child on the basis of “race, colour, sex, language, religion, political or other opinion, national ethnic or social origin, property, disability, birth or other status”(122). Further international legislation obligates to non-discrimination based on gender, such as the Yogyakarta principles plus 10 (YP+10)(123). Although the YP+10 includes children, e.g. Principle 32, it is unclear how this applies to UAMs in practice. Since the 1990s, sexual orientation and gender identity have been included as grounds for asylum under the 1951 UN Convention on the Status of Refugees(124,125). However, adolescents may be viewed as not fully aware of their sexuality, challenging asylum procedures based on sexual orientation and gender identity(126). Government measures that criminalise UAMs by treating them as “illegal” or deny them protections afforded to domestic children clearly violate the non-discrimination prohibition. Another core CRC principle is the ‘best interests’ that requires states, “in all actions concerning children whether undertaken by public or private” bodies, to ensure that “the best interests of the child shall be a primary consideration”(122). This means that policies that treat UAMs as migrants first – excludable at the border, subject to detention, ineligible for welfare services – and children second, are challengeable. Many jurisdictions, including the EU, have included child protection as part of their migration control – access to guardians, child-friendly shelter, specialist staff training - flowing from the best interests principle(127).

CRC Article 22 obliges states to ensure that refugee or asylum seeking children should “receive appropriate protection and humanitarian assistance in the enjoyment of applicable rights…”. Among “applicable rights” are those set out in the 1951 UN Convention on the Status of Refugees as amended by the 1967 Protocol, including protection from “refoulement” (122): UAMs cannot be forcibly expelled to a place where they face life-threatening risks. It is also the state’s obligation to avoid family separation against the child’s will. Measures that indiscriminately impose such expulsions or separations on UAMs violate core humanitarian principles. There is evidence of state authorities justifying restriction on rights on the basis of health emergencies or terrorism, disregarding the *Siracusa Principles* that allow strictly evidence-based, proportional and legal restrictions in the name of public health(128). These measures further marginalize irregular migrant groups such as UAMs. Finally, all children have the right to have their birth registered and to acquire a nationality. Stateless children, including UAMs, may suffer acutely from violation of these fundamental legal identity rights, facing challenges securing eligibility documents for mobility, state services and protection. Health professionals involved in maternity services have a significant role to play in ensuring that the relevant state authorities implement their obligations in this domain.

## Conclusion and limitations

This review aimed to outline all aspects relevant for advocacy of better UAM health. While we give a global perspective, intricacies related to specific contexts remain fundamental to address local capacity building. We did not seek to map practices and intervention strategies – a potential focus for further research. Most limiting is the fact that the evidence the review provides is non-exhaustive, given the lack of comprehensive UAM migration and health data.

Child irregular migration is a complex topic that explicitly shows the brutality of migration enforcement, structural racism and violence. Inadequate data on UAM migration may reflect denial of governments to take full responsibility for their health, as well as failure of investment in health and child protection as critical components of migration governance. Governments should admit their own contribution to migration - by investing in wars, low-wage labour outsourcing, resource exploitation and climate change - instead of manipulating migration ‘numbers’ in xenophobic discourses to the host population. Currently, child protection systems are unsuitable, as two of the most common problems that occur with UAMs – age disputes and disappearances (see panel 1)- remain without apparent solution. A joint effort to orient advocacy, politics, investment and research towards single, resilient and efficient health, education and welfare systems that can benefit host and migrant populations is more critical than ever and can pave the path for global justice. Child health professionals have a crucial role in advocating for improvements in health and the determinants of health. The following section provides recommendations to outline what the objectives of this advocacy should be.

## Recommendations

Recommendations from this review article are based on evidence provided, binding legal obligations and child protection principles. To challenge indifference to migrant and child rights, usually driven by competing economic interests under the name of national security, means challenging the xenophobic and manipulated discourse that conveys recommendations for these rights as charity at best and theft of resources at worst. While it is beyond the scope of this review to assess the historical, social and economic circumstances that drive forced migration, the complexity and the roots of this phenomenon remain critical to understanding and planning for action.

We propose three specific recommendations that are interconnected and are based on the principle that children are children, regardless of nationality. Firstly, non-discrimination in access to healthcare must be a priority, given the long-term consequences that migration can have on children and adolescents. Reaching UAMs means guaranteeing school and health service access, including transportation and information availability, as well as appropriate training of teachers and health professionals(129). Safe accommodation, basic services and the appointment of a guardian, while family reunification checks are done, are essential in guaranteeing UAM health and wellbeing. Health and mental health care access for UAMs must be made routinely available in migratory checkpoints and through mobile health clinics, including sexual and reproductive health services for victims of sexual violence. Clear information campaigns on how to access health services are also required.

Secondly, we need adequate, good quality and ethically collected data that can accurately reflect UAM migration. Indeed, ethical and data protection frameworks should govern the collection, use, sharing, reporting and dissemination of migration health related data, in particular for UAMs. Part of this robust framework should include direct interactions with UAMs that form the evidence base; age and gender disaggregation; standardisation of definitions – including definitions on violence against children(130) – and incentives for national data collection on UAMs. By incentives we mean concrete financial resource commitment (both at UN system and national level) to prioritising children’s healthy development, including appropriately trained staff, and coordination between immigration, child protection and health authorities at national level(131). Specific methods for data collection are available in existing toolkits(132,133). Excellent guidelines also exist for holistic age assessment identification procedures(134,135). Agencies capturing data on UAMs must have robust training and firewalls that ensure data cannot be used by border enforcement purposes. Training should focus on individualised, gender-sensitive and child friendly processing, emphasising the right to be heard. Ideally, a separate body of migration officials should be appointed to deal only with children and/or vulnerable migrants.

Thirdly, UAMs must be explicitly included in national migration and health policies, following international treaties, to guarantee protection of their best interests. UAMs should be managed through their inclusion in domestic child welfare systems, in communication with migration authorities: alternatives to detention must happen. Deportation and ‘voluntary’ return should be assessed not just on the basis of immediate risks but also from a health and mental health perspective, including stability and long-term development(136). Standardisation of reception and care of UAMs should be shared between countries, anticipating influxes with appropriate humanitarian corridors and resettlement incentives that include support of local communities. Predictive analytics – the use of existing data to predict events - aids this anticipation of humanitarian need and is already in use by the UN Centre for Humanitarian Data(137). This should be included in governments’ budget planning, policy implementation and monitoring, with accountability over the use of resources to avoid corruption. Policy should be oriented towards integration of UAMs, through investment in resilient health and education systems which benefit both host and migrant young persons. As UAMs are mostly older adolescents, educational access should include higher education or vocational training. With appropriate measures oriented towards inclusion - such as language classes and activities to combat prejudice - this can prove beneficial for the host country’s economy in the long-term. Finally, child health professionals must be included when both migration and health policies are developed.

## Panel 1: Case Studies

### Paris, France

Since 2017, Médecins Sans Frontières (MSF) has provided medical, psychological, legal and social assistance to more than 1,700 UAMs in France. X is one of them, from Côte d'Ivoire and in France since 2018. During his migration, he suffered sexual violence in Libya and became infected with HIV.

As the protocol of care in France was likely to be compromised by the absence of a legal representative, X appealed to the juvenile judge in Nanterre, who temporarily placed him under protection of Child Protection Services (ASE – Aide Sociale à l’Enfance) of the Hauts-de-Seine department and requested a bone examination to confirm his alleged minority status. Since the result of the assessment did not prove minor age, the juvenile judge ordered the release of the provisional placement measure. Housed by MSF during confinement (March-May 2020), he contracted COVID-19, and was severely clinically decompensated. Admitted to a hospital in the department of Seine-Saint-Denis, he requested the reopening of his file before the Juvenile Court Judge of Nanterre. The Juvenile Judge refused to reopen the case. The hospital then forwarded a notification of worrying situation to the Departmental Council of Seine-Saint-Denis, which ignored the request since X had originally been evaluated in another department. The exhaustion of traditional means of recourse and X's medical situation led MSF to file a complaint on July 1st 2020 against the Departmental Council of Seine-Saint-Denis on the grounds of abandonment of a person who is unable to protect himself (Penal Code, art. 223-3); endangerment of the life of a person (Penal Code, art. 223-1) and failure to assist a person in danger (Penal Code, art. 223-6) due to lack of follow up. The complaint is awaiting a decision from the Public Prosecutor of the Republic. This case reveals the dysfunction of the Child Protection Services and the neglect of these minors even when vulnerability is established.

### Mexico City, Mexico

In Mexico, few spaces exist to specifically look after UAMs as alternatives to detention. CAFEMIN, a migrant shelter in Mexico City, is one of them, where different strategies have been developed to guarantee access to basic services and protection to the best interests of the child in this complex setting. On average during pre-pandemic times, CAFEMIN received 15 to 20 UAMs per month, with a stay of 1-3 months in case of ‘voluntary’ returns and up to a year for those seeking regularisation in Mexico. Every UAM in Mexico is under legal representation by the Child Protection Authority (Procuraduría) for their regularisation, while their care is under the responsibility of the national child welfare (DIF). However, uncertainty and long waiting times mean that these minors often decide to escape the shelters and continue their journey north or back home, giving up their process of asylum in Mexico. In fact, access to mental health services is one of the key aspects of care for UAMs and CAFEMIN works with psychiatry and psychology teams to help. Access to health services has had a better response when the minor is accompanied by CAFEMIN staff, rather than going alone. For example, the complex case of a 17 year old Honduran girl who suffered a psychotic crisis and had to be hospitalised; or a boy who broke his arm and needed surgery and later rehabilitation. Although reception and care for UAMs in Mexico needs to be strengthened, the presence of appropriate legislation and the efforts of civil society spaces like CAFEMIN are steps towards a better future for every child.

## Panel 2: Comparative policy analysis

Policies’ effects are a product of the perspective and interests that inform its development. How a policy context is framed is critical to understanding the pathways through which policy emerges(138). In this sense, policies are not neutral, but are anchored to power dynamics(138). In order to understand the impact current policies have on UAMs globally, a deeper exploration of policy assumptions in this area is needed. From an initial policy screening of six countries representative of the main UAM migration corridors (in Appendix), we selected policies from Turkey - an important transit route to Europe(6)- and Uganda -host of high numbers of refugee UAMs(25). Our analysis is guided by Bacchi’s 'What's the Problem Represented to be?' (WPR) method(139).

For the past six years, Turkey has hosted the largest population of refugees and asylum seekers in the world(140). Turkey’s conceptualisation of migrants as both a threat and potential opportunity to leverage political power is anchored to complex historical-tensions(141). Rising strains in the Turkey-EU relationship in-2020 led to Turkish authorities threatening to ‘open the gates’(142), while on the other side of its territory, along the border with Syria, a 764 kilometre long concrete, razor-wire topped security wall was completed by 2018(143). UAMs’ enhanced level of vulnerability is recognised and provision of care is shared between International Protection and Turkey’s Child Protection Law led by the Ministry of Family, Labour & Social Services. However, in analysis of Turkish law 6458 of 2013 on Foreigners and International Protection (amended 29 Oct 2016)(144), UAMs seeking international protection remain within wider conceptualisation of migrants as potential threats to national security, with burden on them to prove their ‘innocence’(144). Though policy states that UAMs should be treated in line with the best interests of the child(144), no government documentation of how this occurs or the number of UAMs under state care could be detected. Minors under 12 years should enter a child protection institution, while those aged 13-18 years should be in child protection units within refugee camps(145). Those deemed ineligible to remain may also be accommodated in removal centres(144). Whilst Child Protection Law in immigration law emphasises the duty to support children’s needs, the majority of the document details the response to juveniles involved in crime(146). Juvenile support ends at the age of 18, with no information detailing assimilation of UAMs into Turkish society.

Uganda’s refugee policy has been praised for its focus on integration and respect(147). Its 2019-2024 Health Sector Integrated Refugee Response Plan (HSIRRP)(148) reflects commitment to international agreements that foster solidarity between countries. The HSIRRP highlights that refugees should have freedom of movement and access to services as nationals. The problem here is framed as, on one hand, the high number of refugees - 1.1 million at the time of policy writing. On the other hand, “a parallel health system for refugees is unsustainable and promotes inequitable access to health”. The policy proposes one single, resilient, State-led healthcare system, accessible by refugees and nationals, as a sustainable, more financially efficient approach. Implementation requires workforce and infrastructure investment and the policy acknowledges this through inclusion of NGOs and the private sector to fill funding and monitoring gaps. Accreditation for refugee healthcare workers is proposed, allowing refugees to be self-reliant. The assumptions underlying this policy are based on health as a human right, regardless of nationality. However, the assumption of equality in a group with varied needs and levels of access, such as UAMs among children, is left unproblematised. While there is a brief mention of separate service packages for adults and children, UAMs are not mentioned as a special category despite Uganda hosting the second largest number of UAM refugees in the world (40,000 in 2019(25)). The result of this gap is that young people who travel alone may not be able to access these benefits.

The above policies represent alternative framings of migrants and, consequently, UAMs. Whilst Turkey continues to play a global role in hosting large numbers of refugees, conceptualising UAMs as potential societal threats risks devaluing their status as holders of universal and child human rights,with impacts on health. Despite challenges in implementation, Uganda’s representation creates the possibility for UAMs to be included in the country in positive ways that enhance health and wellbeing.

## Search strategy and selection criteria

We conducted searches on PubMed, Embase, PsychINFO and Google Scholar, integrated with searches on migration data portals, government websites, NGO reports and country reports. The combined searches for these sections led to the inclusion of 125 references in the review and additional 23 references were added by co-authors.

## Review of health outcomes

We performed searches on PubMed, Embase, PsychINFO and Google Scholar restricting the publication date from 2000 to 2020. Last date of search: 16/09/2020. For reviews of physical health/morbidity profiles we combine the following keywords: (unaccompanied or separated) AND (minor* or child* or adolescent*) AND (migration or migration transit) AND (health or health profile or health need) OR (mental health or depression or anxiety or post-traumatic stress disorder or substance or schizophrenia) OR (risk* or violence or abuse or trafficking or exploitation). Two reviews were excluded because they did not provide a list of the reviewed articles, another was excluded because it included youth within a wider age range (up to 29 years). Additional references were added by searching reference lists and from recommendations.

Inclusion criteria: review studies describing physical health, morbidity profiles or mental health that included unaccompanied minors as population.

Exclusion criteria: the health profile of unaccompanied minors was not the main focus or they were not included as disaggregated population; the review was included in another review.

## Figures and Tables

**Table 1:** Umbrella review of UAM physical and mental health literature

Reference and article type	Location	Population	Reported findings
Baauw et al, 2019 (90) Systematic review and meta-analysis of 53 studies	USA (26 studies), Europe (13), Australia (8), Canada (4), New Zealand (2)	Refugee children from Africa, Asia or the Middle East n = 223 037	The review found high estimated prevalence rates for: anaemia (prevalence of 14%, from all region of origins; 21·7% Africa; 14·1% Asia; 5% Middle East) haemoglobinopathies (all regions 4%, Africa 7·3%, Asia 16%, Middle East 0·1%) chronic hepatitis B (all regions 3%, Africa 4·5%, Asia 3·3%, Middle East 0·1%) latent tuberculosis infection (all regions 11%, Africa 10·2%, Asia 12·4%, Middle East 4·7%) intestinal infections (all regions 31%, Africa 60·6%, Asia 32·2%, Middle East 20·8%) vitamin D deficiency (all regions 45%, Africa 54·1%, Asia 42·4%, Middle East 70·1%)
Curtis et al, 2018(89) Systematic review of 47 studies	UK (12 studies), Netherlands (7), Spain (6), Sweden (5), Belgium (4), Norway (3), Portugal (2), Scotland (2), Denmark (2), Germany (2), Italy (2), Austria (2), Switzerland (1), Greece (1), Iceland (1), Ireland (1), US (1)	Children < 18 years who had migrated across national borders into, or within, Europe. 20 studies included unaccompanied minors.	Lower risk of engaging in binge drinking, tobacco and cannabis use was seen for migrants from Islamic-majority countries Within lower income migrant families, a transition to processed, energy-dense foods was reported. UAMs are at greater risk of PTSD than accompanied children. Not all migrant children experience poor mental health outcomes.
Kadir et al, 2019 (91) Narrative review of 45 original and review articles	Belgium (4 studies), Germany (4), UK (4), Denmark (3), Sweden (3), Greece (2), Italy (1), Netherlands (1), Norway (1), Austria (1), Spain (1)	Asylum seeking, refugee and undocumented children	Communicable diseases: low vaccination coverage and low immunity to vaccine preventable diseases latent/active TB, malaria, hepatitis B/C, syphilis, Human-T-Lymphotrophic virus types 1/2. Non-communicable diseases: Physical trauma related to migrating (skin/tendon lacerations, fractures, muscle contusions). If left untreated, injuries may become infected Nutritional deficiencies; prevalence of iron deficiency anaemia 4-18%. Dental problems; highest prevalence 65% among UK migrant children.
Kien et al, 2019 (92) Systematic review of 47 studies covered in 53 articles	Germany (8 studies), Denmark (6), Sweden (6), UK (5), Netherlands (4), Norway (4), Belgium (3), Croatia (3), Italy (3), Turkey (3), Austria (1), Finland (1), Greece (1), Slovenia (1)	Unaccompanie d or accompanied asylumseeking children and adolescents or refugee minors (≤21 years, to allow for some outliers in the age group) n= 24,786	Results varied widely among studies. PTSD prevalence 19-52·7%; depression 10·3-32·8%; anxiety 8·7- 31·6%; emotional and behavioural problems 19·8-35% Higher prevalence of PTSD, depression, anxiety among UAMs compared to accompanied minors Most frequent pre- migration stresses among UAMs: separation from or death of family members, armed conflicts, personal threats
Mitra et al, 2019 (93) Review of 13 studies	UK (5 studies), Netherlands (3), USA (2), Germany (2), Norway (1)	Unaccompanie d asylumseeking children	UASC in supportive living arrangements (e.g. foster care) had lower risk of PTSD and depression compared with those in semi-independent care arrangements. One metaanalysis found a benefit of foster care with effect size of 0·3 UASC living in reception settings that restricted freedom had more anxiety symptoms. UASC were less likely than accompanied children to access mental health services and/or receive treatment, e.g. one study found that although 60% of minors reported needing mental healthcare, only 12% received any
Mohwinkel et al, 2018 (94) Systematic review of 9 studies	Norway (3 studies), Netherlands (3), UK (1), Belgium (1), Norway (1)	Unaccompanie d refugee minors	Female URMs were found more affected by post-traumatic or depressive symptoms than their male counterparts. One study found an OR=1.64, p<0.1 for girls. There is only weak evidence regarding other mental health outcomes..
Safi et al, 2017 (95) Systematic review of 20 studies	Not specified	Child and adolescent refugees and asylum seekers	Most prevalent psychiatric disorders: PTSD, depression PTSD rate directly related to number of traumatic events experienced Most children who had guardians could receive their resettlement permit Safety feeling in school, religious belief and commitment in society reduce risk of PTSD, depression and anxiety
von Werthern et al, 2019 (96) Review of 31 studies	UK (7 studies), Norway (6), Belgium (6), Netherlands (5), Austria (2), 1 Sweden (2), Philippines (1), USA (1), Germany (1) Finland (1), Italy (1)	Unaccompanied refugee minors	URMs are at risk of negative mental health developments; adolescence and being female further increase risk PTSD prevalence 17-85%, depression 12·7-76%, anxiety 10·8-85%

## Key Messages

- Unaccompanied minors (UAMs) are a group of migrants who travel without legal guardians. They are more exposed to migration-related risks leading to poor health
- There is lack of comprehensive data on UAMs, especially female, disabled or LGBTQ+, partly due to difficulties in identification and increased undercover migration due to border closures
- Health outcomes for UAMs include nutritional deficiencies, dental caries, skin and gastrointestinal infections, low vaccination coverage, post-traumatic stress disorder, depression and anxiety
- Protective Childhood Experiences (PCEs) such as belonging, appropriate accommodation, health and educational schemes are fundamental in reverting long term, adverse health consequences
- Child health professionals have a valuable role in advocating for better UAM health and in using an evidence-based approach in their clinical care
